# Role of Ryanodine and NMDA Receptors in Tetrabromobisphenol A-Induced Calcium Imbalance and Cytotoxicity in Primary Cultures of Rat Cerebellar Granule Cells

**DOI:** 10.1007/s12640-015-9546-8

**Published:** 2015-07-28

**Authors:** Elzbieta Zieminska, Aleksandra Stafiej, Beata Toczylowska, Jan Albrecht, Jerzy W. Lazarewicz

**Affiliations:** Department of Neurochemistry, Mossakowski Medical Research Centre, Polish Academy of Sciences, Pawinskiego 5, 02-106 Warsaw, Poland; Institute of Biochemistry and Biophysics, Polish Academy of Sciences, Pawinskiego 5A, 02-106 Warsaw, Poland; Nalecz Institute of Biocybernetics and Biomedical Engineering, Polish Academy of Sciences, Trojdena 4, 02-109 Warsaw, Poland; Department of Neurotoxicology, Mossakowski Medical Research Centre, Polish Academy of Sciences, Pawinskiego 5, 02-106 Warsaw, Poland

**Keywords:** Brominated flame retardants, Calcium release, Neuronal culture, Excitotoxicity

## Abstract

The study assessed the role of ryanodine receptors (RyRs) and NMDA receptors (NMDARs) in the Ca^2+^ transients and cytotoxicity induced in neurons by the brominated flame retardant tetrabromobisphenol A (TBBPA). Primary cultures of rat cerebellar granule cells (CGC) were exposed to 7.5, 10, or 25 µM TBBPA for 30 min, and cell viability was assessed after 24 h. Moreover, ^45^Ca uptake was measured, and changes in the intracellular Ca^2+^ concentration ([Ca^2+^]_i_) were studied using the fluo-3 probe. The involvement of NMDARs and RyRs was verified using the pertinent receptor antagonists, 0.5 µM MK-801 and 2.5 µM bastadin 12, which was co-applied with 200 µM ryanodine, respectively. The results show that TBBPA concentration-dependently induces an increase in [Ca^2+^]_i_. This effect was partly suppressed by the inhibitors of RyRs and NMDARs when administered separately, and completely abrogated by their combined application. A concentration-dependent activation of ^45^Ca uptake by TBBPA was prevented by MK-801 but not by RyR inhibitors. Application of ≥10 µM TBBPA concentration-dependently reduced neuronal viability, and this effect was only partially and to an equal degree reduced by NMDAR and RyR antagonists given either separately or in combination. Our results directly demonstrate that both the RyR-mediated release of intracellular Ca^2+^ and the NMDAR-mediated influx of Ca^2+^ into neurons participate in the mechanism of TBBPA-induced Ca^2+^ imbalance in CGC and play a significant, albeit not exclusive, role in the mechanisms of TBBPA cytotoxicity.

## Introduction

Tetrabromobisphenol A (TBBPA) belongs to a large group of brominated flame retardants (BFRs) that are heat-resistant and decrease the chance of ignition of electronic equipment, textiles, and building materials. The economic profits of the use of BFRs are overshadowed by potential environmental and toxicological hazards (Alaee and Wenning 2002; de Wit 2002; Williams and DeSesso 2010; Hendriks et al. 2014). Several studies have demonstrated pharmacological and toxic properties of TBBPA, including endocrine-disrupting activity, immunotoxicity, and neurotoxicity (Kitamura et al. 2005; Nakajima et al. 2009; Reistad et al. 2005, 2007; Hendriks et al. 2014).

Several putative mechanisms of TBBPA neurotoxicity have been proposed. TBBPA inhibits the activity of nicotinic acetylcholine receptors and stimulates GABA receptors (Hendriks et al. 2012); moreover, TBBPA modulates intracellular signaling independently of cell membrane receptor activation. TBBPA interferes with the activities of MAP and PKC kinases and of the ligand-activated transcription factor PPAR-γ, activates caspases, induces enhanced production of reactive oxygen species and β-amyloid and increases the intracellular concentration of Ca^2+^ ([Ca^2+^]_i_) (Canesi et al. 2005; Mariussen and Fonnum 2003; Nakajima et al. 2009; Ogunbayo et al. 2008; Reistad et al. 2005, 2007; Al-Mousa and Michelangeli 2012, 2014; Wojtowicz et al. 2014). In this study, we have focused on the latter phenomenon.

Reistad et al. (2007) described an increase in [Ca^2+^]_i_ and cytotoxicity in primary neuronal cultures challenged with TBBPA. The latter phenomenon was prevented by antagonists of NMDA receptors (NMDARs), which suggested that glutamate receptors can mediate these processes. Using the TM4 Sertoli cell line model, Ogunbayo et al. (2008) showed TBBPA-mediated mobilization of intracellular Ca^2+^ stores via ryanodine receptors (RyRs) and proposed a role of this effect in TBBPA cytotoxicity. Other studies revealed that in addition to intracellular Ca^2+^ release, TBBPA strongly inhibits the sarcoplasmic-endoplasmic reticulum Ca^2+^-ATPase (SERCA) and voltage-sensitive Ca^2+^ channels (Ogunbayo and Michelangeli 2007; Al-Mousa and Michelangeli 2012, 2014; Hendriks et al. 2012, 2014).

Recently, we observed similarity in the Ca^2+^ release induced by 10 µM TBBPA and 0.2 µM thapsigargin in primary cultures of rat cerebellar granule cells (CGC) (Zieminska et al. 2014b). Both TBBPA- and thapsigargin-evoked Ca^2+^ transients were sensitive to inhibition by 200 µM ryanodine as long as the brominated tyrosine derivative bastadin 12 was present at a concentration of 2.5 µM, whereas ryanodine alone had a weak or no effect (Zieminska et al. 2014b).

Ryanodine is a selective ligand of RyRs, bidirectionally interfering with RyRs in a concentration-dependent way. At submicromolar concentrations, ryanodine binding results in half-opening of the receptor channel, leading to Ca^2+^ efflux from ER to the cytoplasm and to transient rise in $[{\text{Ca}}^{2+}]_{{\text{i}}}$, while at micromolar concentrations ryanodine closes RyRs (Fill and Copello 2002). Bastadins are cyclic derivatives of brominated tyrosine; many biologically active bastadins interfere with the formation of RyR-immunophilin FKBP12 complexes (Mack et al. 1994; Chen et al. 1999). Recently, we demonstrated that in CGC, the increase in [Ca^2+^]_i_ induced by 10 µM TBBPA is resistant to inhibition by 200 µM ryanodine, whereas its co-administration with 2.5 µM bastadin 12, which by itself has no effect on the level of Ca^2+^, completely inhibited TBBPA-evoked calcium transients (Zieminska et al. 2014b). Importantly, the combination of bastadin 5 or bastadin 12 with ryanodine also inhibits the intracellular Ca^2+^ release induced by thapsigargin or non-coplanar 2,2′,3,5′,6-pentachlorobiphenyl (PCB 95) (Mack et al. 1994; Gafni et al. 2004; Pessah et al. 2010; Zieminska et al. 2006a, b). These phenomena have been attributed to the conversion by these Ca^2+^ releasers of ryanodine-sensitive RyRs into leak channels, which are insensitive to ryanodine. In contrast, bastadins at low micromolar concentrations reverse this phenomenon and restore the sensitivity of RyRs to ryanodine (Pessah et al. 1997; Eltit et al. 2010; Altamirano et al. 2012).

While excessive mobilization of Ca^2+^ from endoplasmic reticulum stores by thapsigargin and other Ca^2+^ releasers has been implicated in their neurotoxicity (Silverstein and Nelson 1992; Pessah et al. 2010; Stirling et al. 2014), contrasting reports have appeared envisaging neuroprotective potential residing in the up-regulation of RyRs and in the ensuing increased release of Ca^2+^ from the ER (Supnet et al. 2010).These discrepant effects prompted us to consider bastadin 12 and ryanodine as pharmacological tools to get a deeper insight into the role of intracellular Ca^2+^ release by TBBPA via RyRs in cytotoxicity, which to our knowledge has not been directly tested in CGC or neurons cultured from any other brain region. In addition, we considered the putative role of NMDARs in the TBBPA-induced increases in [Ca^2+^]_i_ in neurons by triggering the influx of extracellular Ca^2+^, resulting in cytotoxicity. The role of NMDARs and Ca^2+^ in the mechanism of neuronal excitotoxicity is well known and the neuroprotective potential of MK-801 under these conditions has since long been recognized (Choi 1985; Szydlowska and Tymianski 2010).

The aim of the present study was to assess the role of Ca^2+^ release from intracellular stores and its influx via NMDARs in the mechanisms of TBBPA-induced Ca^2+^ imbalance and toxicity in neurons. In this study, we investigated the effects of the acute application of 7.5, 10, and 25 µM TBBPA on changes in [Ca^2+^]_i_, ^45^Ca uptake from the incubation medium and cell survival in primary cultures of rat CGC. The modulators of the effects of TBBPA used in this study included the NMDAR antagonist MK-801 and the RyR antagonist ryanodine which was co-applied with bastadin 12.

## Materials and Methods

### Reagents

Tetrabromobisphenol A (2,2-bis(4-hydroxy-3,5-dibromophenyl)propane, TBBPA) and 2,2′,3,5′,6-pentachlorobiphenyl (PCB 95) were obtained from LCG Standards Poland (Lomianki, Poland). l-Glutamate, ryanodine, (+)-5-methyl-10,11-dihydro-5H-dibenzo[a,d]·cyclohepten-5,10-imine hydrogen maleate (MK-801), dimethyl sulfoxide (DMSO), and propidium iodide, which were of analytical grade, as well as fetal bovine serum, cytosine β-d-arabinofuranoside, and the other materials for cell culturing were purchased from Sigma-Aldrich Chemical Poland (Poznań, Poland). Bastadin 12, which was provided by Dr. Emmanuel N. Pitsinos (Laboratory of Natural Products Synthesis and Bioorganic Chemistry, Institute of Bioorganic Chemistry NCRS “Demokritos” in Athens, Greece), was synthesized there according to the method of Couladouros et al. (2005). Fluo-3AM was purchased from Molecular Probes Inc. (Paisley, UK). ^45^CaCl_2_ was obtained from by the National Centre for Nuclear Research, Radioisotope Centre POLATOM (Otwock, Poland). All the other chemicals were of analytical grade.

### Animals and Cell Culture

Primary neuronal cultures were prepared from 7-day-old Wistar rats. For these experiments, we used rat pups, which were obtained from the animal colony of the Mossakowski Medical Research Centre, Polish Academy of Sciences in Warsaw. Mothers and pups were fed and watered ad libitum and kept on a 12:12 h dark-light cycle, at room temperature with a constant humidity of approximately 60 %. The procedures were approved by the Fourth Local Ethical Committee in Warsaw and were performed in accordance with EC Directive 86/609/EEC of 24 November 1986. We made all efforts to reduce the number of animals used and to minimize their suffering.

CGC were isolated and cultured according to a standard method (Schousboe et al. 1985), as described elsewhere (Zieminska et al. 2006a, 2012, 2014a, b). Briefly, the cerebella were chopped and trypsinized. Then, the cellular suspension obtained by trituration was centrifuged. The cells were re-suspended in Eagle’s basal medium supplemented with 10 % fetal calf serum, 25 mM KCl, 4 mM glutamine, streptomycin (50 μg/ml), and penicillin (50 U/ml) and were seeded at a density of 1, 2 or 4 × 10^6^ cells per well on 24-,12- or 6-well plates (Thermo Scientific™ Nunc™) coated with poly-l-lysine. After 48 h of culturing, 7.5 μM cytosine arabinofuranoside was applied to inhibit DNA replication and the growth of non-neuronal cells. The experiments were performed after 7 days in vitro.

### Measurement of Changes in [Ca^2+^]_i_

**Fig. 1:**
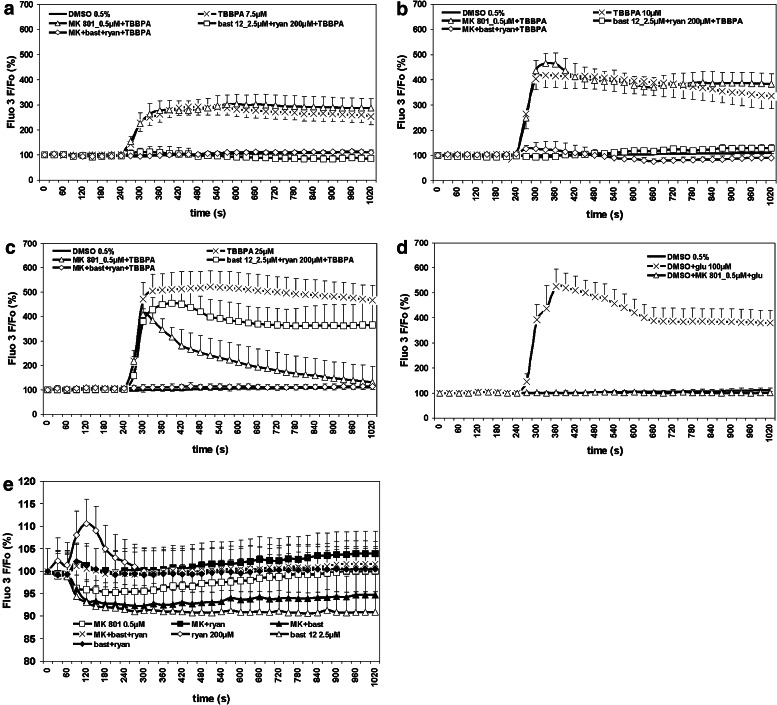
TBBPA- and glutamate-induced changes in intracellular Ca^2+^ concentration in primary cultures of rat CGC measured with a confocal fluorescence microscope. The effects of TBBPA at concentrations of 7.5 µM (**a**), 10 µM (**b**) and 25 µM (**c**) or of 100 µM glutamate (**d**) on the fluorescence of the fluo-3-loaded cells were measured in Locke 5 incubation medium containing, as indicated, vehicle (0.5 % DMSO), 0.5 µM MK-801, and/or 2.5 µM bastadin 12 (bast 12) with 200 µM ryanodine (ryan). Panel (**e**) shows effects of MK-801, and bastadine 12 plus ryanodine applied without TBBPA. Fluo-3 fluorescence is expressed as a percentage of the basal level (Δ*F*/*F*_0_%). The results are the mean ± SD (*n* = 15). For the significance of differences among the experimental groups, see Table 1

**Fig. 2:**
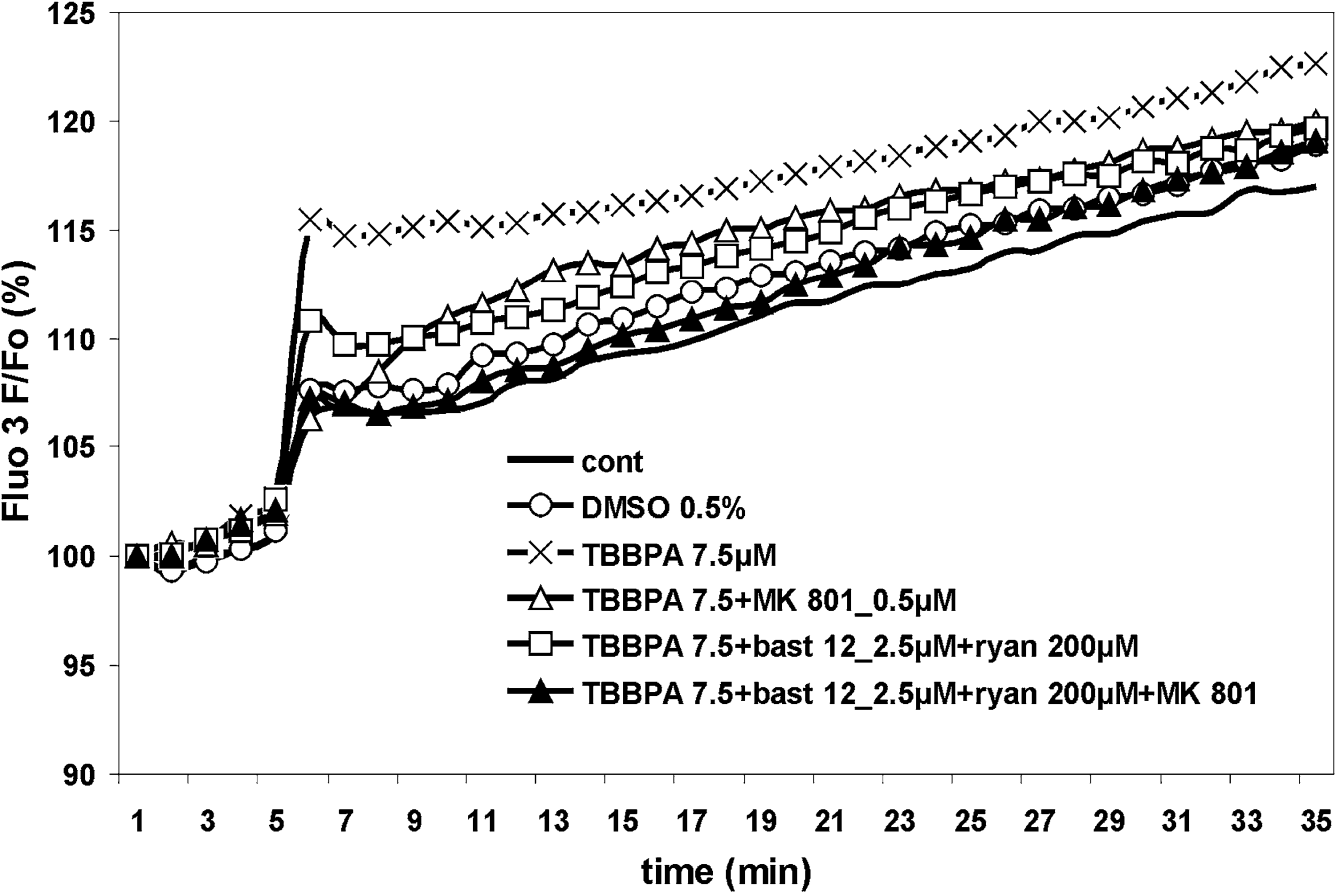
TBBPA-induced changes in intracellular Ca^2+^ concentration in primary cultures of rat CGC, as measured with a fluorescence plate reader. The effects of 7.5 µM TBBPA on the fluorescence of the fluo-3-loaded cultures were measured using pharmacological tools, as described for Fig. 1. Fluo-3 fluorescence is expressed as a percentage of the basal level (Δ*F*/*F*_0_%). The results are the mean ± SD (*n* = 4). For the significance of differences among the experimental groups, see Table 2

Changes in intracellular Ca^2+^ concentration were measured using fluorometric methods exactly as described previously (Zieminska et al. 2014a, b). For the experiments using a confocal fluorescence microscope, the CGC cultures were loaded with the fluorescent calcium-sensitive probe fluo-3 via a 30-min incubation at 37 °C in the original growth medium containing 4 μM fluo-3AM. Next, the growth medium was removed; the cells were washed three times with Locke 5 buffer containing 154 mM NaCl, 5 mM KCl, 4 mM NaHCO_3_, 2.3 mM CaCl_2_, 5 mM HEPES (pH 7.4), and 5 mM glucose and pre-incubated for 5 min in this medium. Then, 0.5 µM MK-801, and/or 2.5 µM bastadin 12 together with 200 µM ryanodine were added for an additional 5 min before application of TBBPA, DMSO, and positive controls glutamate or PCB 95, as presented in Figs. 1 and 5. The fluorescence of the cell-entrapped fluo-3 at 530 nm was measured every 30 s using an LSM 510 confocal microscope equipped with a 488-nm argon laser for the induction of fluorescence and the data acquisition software LSM 510 version 3.2 (Carl Zeiss AG, Jena, Germany). In experiments using a fluorescence plate reader (Fig. 2), CGC cultures (1 × 10^6^ cells per well) were preincubated with 4 μM fluo-3AM. After washing the cultures three times with Locke 5 buffer, fluorescence was measured using a microplate reader (FLUOstar Omega, Germany) set at 485 nm excitation and 538 nm emission wavelengths. Baseline fluorescence was measured, and then the changes in fluorescence after the addition of each of the tested compounds were recorded every 60 s.

There was no need to correct for autofluorescence in either of the two experimental settings, as it was stable and did not exceed 10 % of the basal level of fluorescence of plates with fluo-3-loaded CGC. The results in Figs. 1 and 2 are presented graphically as percentages of the change in fluorescence in relation to the basal level (Δ*F*/*F*_0_%). In addition, quantitative data characterizing the increase in fluo-3 fluorescence above the basal level, measured just after the rapid phase of the Ca^2+^ transient and at the steady-state level, i.e., 60 and 300 s after TBBPA or glutamate application, respectively, and the significance of the differences are presented in Tables 1 and 2. The data in Fig. 1 and Table 1 were obtained in three independent experiments, each of which used a different preparation of CGC cultures. In each experiment, five wells were used per treatment. The results from each well consisted of the average data collected from 15 randomly selected objects: cell bodies or their conglomerates. Because the basal levels of fluorescence and the maximal responses to the positive control (100 µM glutamate) or to corresponding concentrations of TBBPA did not differ significantly among any of these experiments (one-way ANOVA, *p* < 0.05), the corresponding data obtained in separate experiments were combined, analyzed as a single representative set for each treatment and presented as the mean ± SD of 15 repetitions. The data in Fig. 2 and Table 2 (mean ± SD) are the results of one example experiment, in which four wells were used for each treatment. This experiment was repeated three times, and qualitatively similar results were obtained.

**Table 1:** Modulation by antagonists of NMDA and ryanodine receptors of TBBPA- and glutamate-evoked increases in intracellular Ca^2+^ concentration in primary cultures of rat cerebellar granule cells: fluorescence microscopy measurements

Treatment groups & modulating substances (μM)	TBBPA (7.5)	TBBPA (10)	TBBPA (25)	Glutamate (100)
	Rise in fluo-3 fluorescence related to the baseline (%)			
*60 s after TBBPA or glutamate application*				
None	249 ± 31^a^	417 ± 47^a^	505 ± 68^a^	437 ± 71^a^
MK-801 (0.5)	265 ± 41^a^	465 ± 38^a^	385 ± 54^a^	100 ± 5^a^
Bastadin 12 (2.5) + ryanodine (200)	111 ± 21^b^	94 ± 5^b^	428 ± 49^a,b^	n.d.
MK-801 + Bastadin 12 + ryanodine	97 ± 7^b^	123 ± 23^b^	110 ± 11^b^	n.d.
*300 s after TBBPA or glutamate application*				
None	279 ± 29^a^	394 ± 54^a^	511 ± 65^a^	400 ± 54^a^
MK-801 (0.5)	300 ± 41^a^	373 ± 39^a^	202 ± 24^a,b^	102 ± 5^a^
Bastadin 12 (2.5) + ryanodine (200)	92 ± 15^b^	118 ± 7^b^	373 ± 59^a,b^	n.d.
MK-801 + Bastadin 12 + ryanodine	1107 ± 7^b^	80 ± 12^b^	114 ± 11^b^	n.d.

The results of Fig. 1 characterizing the initial rises and steady-state levels of the fluo-3 fluorescence after the application of TBBPA or glutamate. Other information as described in legends to Fig. 1. Results are mean ± SD (*n* = 15)

^a^Results significantly different from the baseline

^b^Results significantly different from the corresponding groups untreated with modulating agents (*p* < 0.05)

**Table 2:** Modulation by antagonists of NMDA and ryanodine receptors of TBBPA-evoked increases in intracellular Ca^2+^ concentration in rat cerebellar granule cells in primary culture: fluorescence plate reader measurements

Treatment groups & modulating substances (μM)	TBBPA (7.5) Rise in fluo-3 fluorescence related to the baseline (%)
*60 s after TBBPA application*	
None	115 ± 15^a^
MK-801 (0.5)	106 ± 7
Bastadin 12 (2.5) + ryanodine (200)	111 ± 8
MK-801 + bastadin 12 + ryanodine	107 ± 7^b^
*300 s after TBBPA application*	
None	115 ± 14^a^
MK-801 (0.5)	112 ± 9^a^
Bastadin 12 (2.5) + ryanodine (200)	111 ± 9
MK-801 + bastadin 12 + ryanodine	109 ± 8

The results of Fig. 2 characterizing the initial rises and steady-state levels of the fluo-3 fluorescence after the application of TBBPA. Other information as described in legends to Fig. 2. Results are mean ± SD (*n* = 4), presenting data from one example experiment

^a^Results significantly different from the baseline

^b^Results significantly different from the corresponding groups untreated with modulating agents (one-way ANOVA test, *p* < 0.05)

### Measurement of ^45^Ca Uptake

**Fig. 3:**
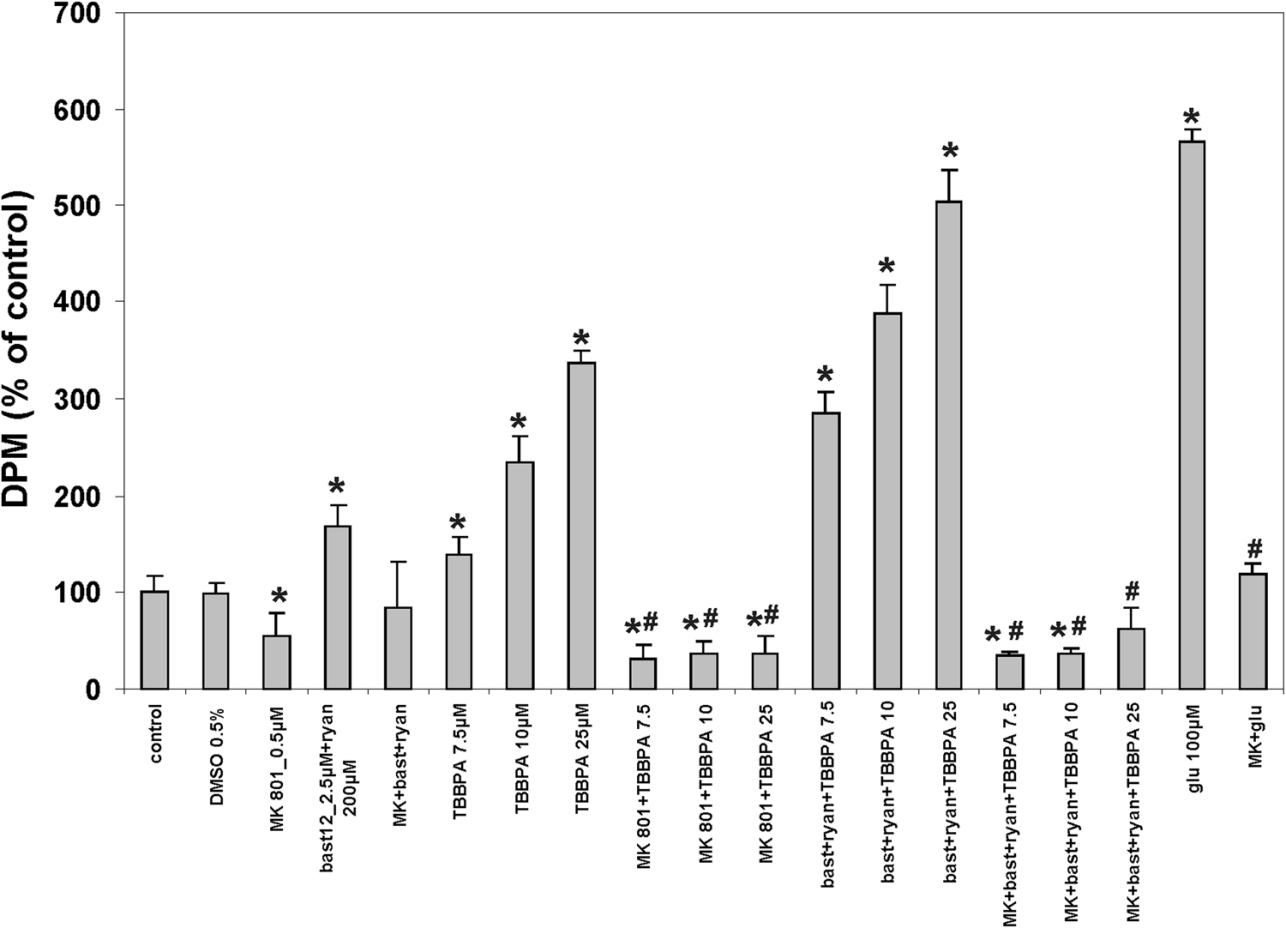
^45^Ca uptake in primary cultures of rat CGC induced by 7.5, 10, and 25 µM TBBPA or by 100 µM glutamate. The cells were incubated in Locke 5 medium containing, as indicated, vehicle (0.5 % DMSO), 0.5 µM MK-801, or 2.5 µM bastadin 12 (bast 12) with 200 µM ryanodine (ryan). The results are the mean ± SD (*n* = 6). *  The effects of TBBPA and glutamate are significantly different from the control groups. # The effects of the inhibitors are significantly different from the corresponding TBBPA- or glutamate-treated groups (*p* < 0.05)

The procedure was basically performed as described previously (Zieminska et al. 2014a). The CGC (4 × 10^6^/well) were pre-incubated for 5 min in the Locke 5 medium, followed by a 5-min incubation without or with 0.5 µM MK-801 and/or 2.5 µM bastadin 12 together with 200 µM ryanodine. Then, 7.5, 10 or 25 µM TBBPA, 0.5 % DMSO (the vehicle control), or else the positive controls 100 µM glutamate or 25 µM PCB 95, were added for 10 min together with ^45^CaCl_2_ (1 μCi/well). The incubation was terminated by washing with ice-cold calcium-free medium containing 2 mM EGTA, and the cells were dissolved in 0.5 M NaOH. The content of radioactive ^45^Ca in the cultures was measured by liquid scintillation spectroscopy using a Wallac 1409 counter (Wallac, Turku, Finland). The data in Figs. 3 and 5 represent the mean ± SD of 6 independent experiments using different CGC preparations. In each experiment, two wells were used per treatment, and the mean values were utilized for further calculations.

### Evaluation of CGC Viability

**Fig. 4:**
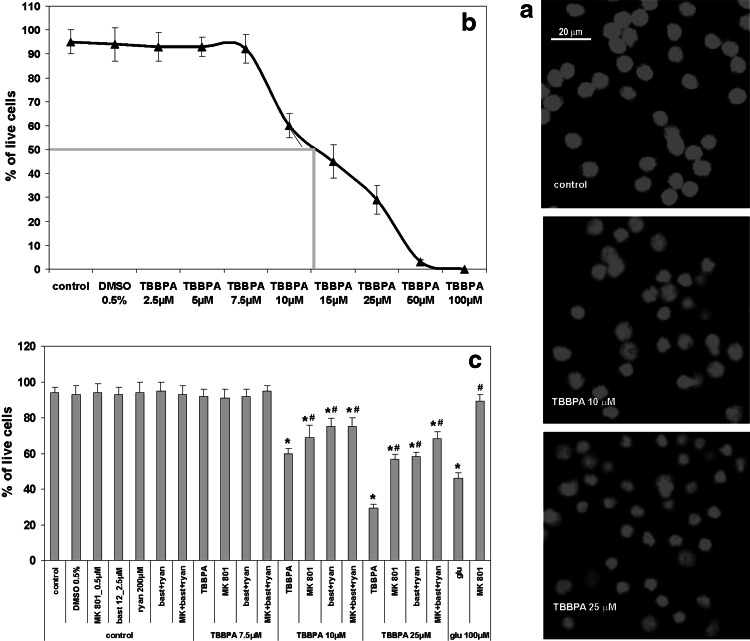
Acute cytotoxicity induced by TBBPA in primary cultures of rat CGC. CGC, control or challenged with 10 and 20 µM TBBPA, stained with propidium iodide (**a**). Evaluation of concentration-dependent acute cytotoxicity induced by TBBPA; the LC_50_ value for TBBPA equal to 12.5 µM (**b**). Toxic effects of 7.5, 10, and 25 µM TBBPA in comparison to that induced by 100 µM glutamate in primary cultures of rat CGC: modulation by 0.5 µM MK-801 and 2.5 µM bastadin 12 plus 200 µM ryanodine (**c**). The cells were incubated for 30 min in the ionic medium containing additions as indicated, and then were cultured in growth medium for 24 h. The percentage of neurons surviving was determined by propidium iodide staining. The results are the mean ± SD (*n* = 6). * The effects of TBBPA are significantly different from the *naïve* control and DMSO (vehicle)-treated cells, and the effects of glutamate (glu) are significantly different from the control cells (*p* < 0.05). ^#^ The effects of MK-801 and bastadin 12 with ryanodine differ significantly from the effects of either TBBPA or glutamate alone (*p* < 0.05)

The experiments were initiated by replacing the growth medium of the CGC cultures (2 × 10^6^ cells per well) with Locke 25 buffer containing 134 mM NaCl, 25 mM KCl, 2.3 mM CaCl_2_, 4 mM NaHCO_3_, 5 mM HEPES (pH 7.4), and 5 mM glucose. As required, freshly prepared solutions of TBBPA or bastadin 12 (both in DMSO), vehicle (0.5 % DMSO), glutamate, or PCB 95 in DMSO (the positive controls), ryanodine, or MK-801 were also included. To evaluate TBBPA or glutamate toxicity, the cells were incubated with these substances at 37 °C for 30 min; then, the exposure was terminated by two washes with Locke 25 buffer, and the CGC were cultured in the original growth medium for an additional 24 h under standard conditions (Ankarcrona et al. 1995, 1996). Finally, the cells were fixed with 80 % methanol and stained with 0.5 µg/ml propidium iodide, and viable and dead neurons were counted using an Axiovert fluorescence microscope (Carl Zeiss AG, Germany) by an investigator who was blind to the experimental conditions, as described previously (Zieminska et al. 2006a). For visualization of changes in staining of nuclei with propidium iodide (Fig. 4a), we used a LSM 510 confocal microscope (Carl Zeiss AG, Germany) equipped with 546 nm HeNe laser for excitation, while pictures were taken at 617 nm emission wavelength. Neuronal viability was expressed as the percentage of live cells in proportion to all cells. For each experiment, the viability of the untreated cells was determined, and the data presented in Fig. 4 were normalized to these control values, which were set as 100 %. Moreover, the other cultures defined in Fig. 4 as the vehicle control were incubated for 30 min in Locke 25 buffer in the presence of 0.5 % DMSO. In each experiment, two wells were used per treatment, and mean values were taken for calculations. The data represent the mean ± SD of 6 independent experiments using different CGC preparations.

### Statistical Analysis

The results are presented as mean ± SD. Differences in corresponding data points between different groups were tested with one-way ANOVA followed by Dunn’s correction method. For all tests, *p* < 0.05 was considered significant. For the statistical analysis, Statistica software (ver. 10, StatSoft) was used.

## Results

### TBBPA-Induced Ca^2+^ Imbalance in CGC

#### Effects of TBBPA on [Ca^2+^]_i_

**Fig. 5:**
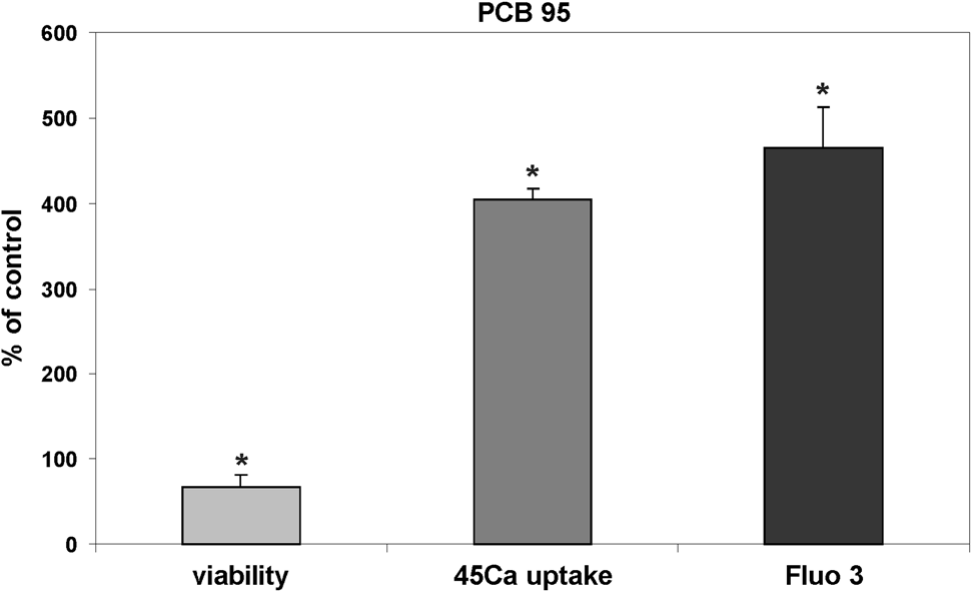
The effect of 25 µM PCB 95 on viability of primary cultures of rat CGC, intracellular Ca^2+^ level, and ^45^Ca uptake. Further information as for Figs. 1, 3, and 4. * The effects of PCB 95 are significantly different from the corresponding control groups

Changes in fluo-3 fluorescence, which are indicative of the alterations in [Ca^2+^]_i_, in the primary CGC cultures are presented in Figs. 1, 2 and 5, and in Tables 1 and 2 corresponding to Figs. 1 and 2. Measurements made with the confocal fluorescence microscope focused on the neuronal cell bodies and their conglomerates revealed that TBBPA applied at 7.5, 10, and 25 µM concentrations induced a rapid, concentration-dependent increase in [Ca^2+^]_i_ to the maximal levels of 292, 417, and 521 % relative to the basal level, respectively, whereas administration of the vehicle, 0.5 % DMSO, did not change basal fluo-3 fluorescence (Fig. 1a–c; Table 1). The maximal increase in [Ca^2+^]_i_ evoked by 25 µM TBBPA was similar in magnitude to the effects of both reference agents. Administration of 25 µM PCB 95 resulted in 465 % increase in [Ca^2+^]_i_ (Fig. 5), while 100 µM glutamate produced a 526 % increase in the intracellular Ca^2+^ level (Fig. 1d).

The NMDAR antagonist MK-801 (0.5 µM), did not interfere with the increases in [Ca^2+^]_i_ induced by 7.5 and 10 µM TBBPA (Fig. 1a, b) but partially reduced a similar effect evoked by 25 µM TBBPA (Fig. 1c; Table 1). The increase in [Ca^2+^]_i_ induced by 100 µM glutamate was completely inhibited by 0.5 µM MK-801 (Fig. 1d). We also evaluated how 2.5 µM bastadin 12 applied together with 200 µM ryanodine, which were previously shown to inhibit the release of intracellular Ca^2+^ induced by 10 µM TBBPA (Zieminska et al. 2014b), interferes with increases in [Ca^2+^]_i_ induced by TBBPA at the tested concentrations. The results of Fig. 1a, b demonstrated that the administration of bastadin 12 together with ryanodine completely inhibited the increases in [Ca^2+^]_i_ induced by 7.5 and 10 µM TBBPA and that the additional application of 0.5 µM MK-801 did not modify this effect (Fig. 1a, b). The increase in [Ca^2+^]_i_ evoked by 25 µM TBBPA was partially reduced by bastadin 12 with ryanodine, whereas the combination of bastadin 12 and ryanodine with MK-801 completely abolished this effect (Fig. 1c; Table 1). As shown in Fig. 1e, application of MK-801, bastadine 12, and ryanodine alone or in combination, but in the absence of TBBPA, produced only minor changes in [Ca^2+^]_i_. In particular, we detected a short-term and a slight increase in [Ca^2+^]_i_ after administration of ryanodine, a phenomenon already characterized in earlier studies (Hernández-Cruz et al. 1997; Zieminska et al. 2014b).

To verify the findings from the fluorescence microscope that MK-801 does not inhibit Ca^2+^ transients induced by TBBPA at low micromolar concentrations, in the next experiments, we examined changes in [Ca^2+^]_i_ evoked by 7.5 µM TBBPA in CGC cultures using a fluorescence plate reader as a platform for measuring fluo-3 fluorescence. In contrast to the experiments using a fluorescence microscope, data from the fluorescence plate reader showed a steady upward trend of F/F_0_% (Fig. 2), which is consistent with the observations of Heusinkveld and Westerink (2011) and Meijer et al. (2014). Control experiments showed no detectable effect of 2.5 µM bastadin 12 or 200 µM ryanodine applied alone on the basal level of [Ca^2+^]_i_ (results not shown). Notwithstanding the slight increase in [Ca^2+^]_i_ evoked by 7.5 µM TBBPA, and a tendency to its partial inhibition by both MK-801 and bastadin 12 plus ryanodine, the application of these substances in combination resulted in a nearly complete inhibition of the Ca^2+^ transients (Fig. 2; Table 2). Qualitative data obtained in repetitive experiments, consistently demonstrated an inhibitory effect of MK-801 on the Ca^2+^ transients evoked by 7.5 µM TBBPA. However, a large degree of variability in the F/F_0_% level noted in individual experiments precluded reasonable presentation of the cumulative results of all of these experiments; therefore, only data from one example experiment are shown (Fig. 2; Table 2).

#### Effects of TBBPA on ^45^Ca Uptake

The results of measurements of ^45^Ca uptake by CGC, which reflects the influx of extracellular Ca^2+^ into the neurons, are presented in Figs. 3 and 5 in relation to the level of ^45^Ca that accumulated in untreated CGC (control). DMSO (0.5 %), the vehicle for TBBPA, bastadin 12, and PCB 95, had no effect on ^45^Ca uptake, whereas an antagonist of NMDARs, 0.5 µM MK-801, reduced basal ^45^Ca uptake by half (Fig. 3). This result reflects the level of ongoing glutamatergic neurotransmission mediated by NMDARs in CGC cultures. Application of the RyR antagonists, 2.5 µM bastadin 12 together with 200 µM ryanodine, potentiated ^45^Ca uptake to 169 % of the control, and this effect was completely abolished in the presence of MK-801 (Fig. 3). In the presence of 7.5 µM TBBPA, the accumulation of radio-labeled Ca^2+^ by the CGC was 138 % higher than the accumulation by the vehicle control-treated cells; it increased to 234 % in the presence of 10 µM TBBPA and to 336 % by 25 µM TBBPA. This concentration-dependent potentiation of ^45^Ca uptake by TBBPA was completely inhibited by MK-801 but, in contrast, was increased in an additive manner by the RyR inhibitors, 2.5 µM bastadin 12 with 200 µM ryanodine. The latter effect was completely abolished by MK-801 (Fig. 3). Hundred µM glutamate, which served as positive control induced more than a five-fold rise in ^45^Ca uptake, which was reduced to almost basal levels by MK-801 (Fig. 3). Treatment with one other reference compound, 25 µM PCB, has likewise increased ^45^Ca uptake (a fourfold increase, Fig. 5).

### Effect of TBBPA on Cell Viability

The results of Figs. 4 and 5 demonstrate the viability of the CGC 24 h after a 30 min incubation in the ionic medium. In control cultures that during such an incubation were not exposed to any pharmacological substances, viability was 94 %. Exposure of the neuronal cultures to 0.5 % DMSO, which was the vehicle of TBBPA, bastadin 12 and PCB 95, had no additional effect on cell viability (Fig. 4b, c). The results of control experiments demonstrated that the exposure of CGC to ryanodine, bastadin 12, or their combination without TBBPA did not influence neuronal viability (Fig. 4c). The same was true for the cells incubated with non-toxic 7.5 µM TBBPA. Glutamate (100 µM), which was applied as a positive control to induce the excitotoxicity that is mediated mainly by NMDARs, produced a pronounced, significant decrease in the percentage of live cells to 46 %. This toxic effect of glutamate was completely reversed in the presence of the NMDAR antagonist 0.5 µM MK-801, which was nontoxic by itself when acutely applied (Fig. 4c). The other positive control PCB 95 (25 µM) reduced viability of CGC to 67 % (Fig. 5).

The data presented in Fig. 4a–c show that a 30 min exposure of CGC to 7.5 µM TBBPA did not reduce the viability of the cells after 24 h, whereas in cultures exposed to higher concentrations of TBBPA, significant cytotoxicity was found. Treatment with 10 µM TBBPA reduced the percentage of live neurons to 60 %, while TBBPA at a concentration of 25 µM led to a decrease of the percentage of live cells to only 29 %. TBBPA applied at 50 and 100 µM concentration killed practically all the neuronal cells. The LC_50_ for TBBPA has been estimated to 12.5 µM (Fig. 4b).

To assess the contribution of NMDARs and RyRs to TBBPA cytotoxicity, we applied 0.5 µM MK-801 or 2.5 µM bastadin 12 and 200 µM ryanodine, respectively. As presented in Fig. 4c, MK-801 did not change the normal high viability of CGC incubated with 7.5 µM TBBPA. MK-801 also slightly increased the percentage of live neurons after incubation with 10 µM TBBPA, to 69 %. In CGC treated with 25 µM TBBPA in the presence of MK-801, the fraction of surviving neurons increased to 57 %. In turn the percentage of live CGC after incubation with 10 µM TBBPA in the presence of ryanodine with bastadin 12 increased to approximately 75 % and in the case of 25 µM TBBPA, the percentage of live CGC increased to 58 % (Fig. 4c). Thus, the application of MK-801 and bastadin 12 with ryanodine provided similar levels of cytoprotection. Moreover, as presented in Fig. 4c, the combination of the antagonists of NMDARs and RyRs had the same cytoprotective effect on CGC treated with 10 µM TBBPA as these antagonists when applied separately. Only in the case of the cytotoxicity induced by 25 µM TBBPA, the application of MK-801, bastadin 12, and ryanodine in combination increased the percentage of live neurons to approximately 68 %, and this level of viability was slightly but significantly (*p* < 0.05) higher than the viability observed after separate application of these antagonists of NMDARs and RyRs (Fig. 4c).

The above experiments revealed that a portion of TBBPA cytotoxicity was resistant to NMDAR and RyR inhibitors and therefore could be classified as independent of Ca^2+^ dyshomeostasis. We speculated that despite the removal of the TBBPA- and inhibitor-enriched ionic medium and extensive washing after the 30-min incubation a portion of the TBBPA could remain associated with the cells, in consequence destabilizing Ca^2+^ homeostasis during the additional 24 h of culturing. To account for this possibility, we performed complementary experiments in which CGC were cultured for 24 h in the presence of MK-801, bastadin 12 and ryanodine after completion of the exposure to TBBPA. The results of these experiments did not differ from the data presented in Fig. 4c, i.e., there was no enhancement of cytoprotection (results not shown), thus invalidating the caveat.

## Discussion

In this study, we tested the roles of intracellular Ca^2+^ release via RyRs and of the influx of extracellular Ca^2+^ to neurons through NMDARs in TBBPA-evoked Ca^2+^ imbalance and cytotoxicity in primary cultures of rat CGC. The results demonstrated that the two mechanisms collectively contribute to the Ca^2+^ transients induced in neurons by TBBPA and subsequently participate in a considerable, but not overwhelming degree, to the cytotoxicity induced by acute challenge with TBBPA at concentrations ≥10 µM.

The primary culture of CGC is an in vitro model of glutamatergic neurons that has been widely used in neurobiological and neurotoxicological experiments (Schousboe et al. 1985; Contestabile 2002). We found this model particularly useful in mechanistic studies concerning the nature of Ca^2+^ transients in neurons and the mechanisms of the cytotoxicity of different compounds (Zieminska et al. 2006a, 2006–2007, 2010, 2012, 2014a, b). Importantly, CGC were utilized in the study of Reistad et al. (2007), which first demonstrated Ca^2+^ transients in neurons challenged with TBBPA at micromolar concentrations and showed that MK-801 protects these neuronal cultures against TBBPA-induced cytotoxicity. Later studies that used cell lines that do not express functional NMDARs drew attention to the TBBPA-induced release of intracellular Ca^2+^ via RyRs (Ogunbayo et al. 2008; Al-Mousa and Michelangeli 2012, 2014; Hendriks et al. 2014). Although our previous studies using CGC also showed TBBPA-induced intracellular Ca^2+^ release that seems to result from transformation of the functional RyRs into leak channels (Zieminska et al. 2012, 2014b), in the present work, we revisited the putative role of glutamate receptors in TBBPA-induced Ca^2+^ transients and cytotoxicity in neurons.

The basic pharmacological tool to verify the role of NMDARs in these experiments was their uncompetitive antagonist, MK-801. MK-801 was intentionally used at the relatively low concentration of 0.5 µM, which effectively inhibits NMDAR channels while avoiding off-target effects; for instance, MK-801 inhibits nicotinic acetylcholine receptor channels with an IC_50_ of as low as 3 µM (Amador and Dani 1991). MK-801 is a high-affinity NMDAR channel blocker; studies of the cytoprotective effects of the enantiomers of MK-801, (+)MK-801, and (−)MK-801, in CGC treated with 100 µM glutamate demonstrated their EC_50_ to be 30 nM and 150 nM, respectively (Lysko et al. 1992). Additionally, the present data (Figs. 1, 3) show that 0.5 µM MK-801 almost completely inhibited glutamate-induced increases in [Ca^2+^]_i_ and ^45^Ca influx in cultured CGC.

Collective analysis of results of [Ca^2+^]_i_ measurements using a fluorescence microscope and a fluorescence plate reader, allows to conclude beyond doubt that NMDARs are involved in the TBBPA-evoked Ca^2+^ imbalance. While confocal fluorescence microscopy data bespeak concentration-dependent increases in [Ca^2+^]_i_ induced by 7.5, 10 and 25 µM TBBPA, only the effect of TBBPA at the highest concentration was partially inhibited by MK-801 (Fig. 1). However, using a fluorescence plate reader, we showed that the partial MK-801-mediated inhibition of the increase in [Ca^2+^]_i_ was already evident for 7.5 µM TBBPA (Fig. 2). In our previous studies, both confocal fluorescence microscopy and the fluorescence microplate readouts have proved useful for measuring changes in [Ca^2+^]_i_ in CGC (Zieminska et al. 2006–2007, 2012, 2014a, b). We attribute the discrepancy of the current results to differences in the resolution of the measurements of fluo-3 fluorescence using the two different platforms. While the measurements using the fluorescence microscope were focused on neuronal cell bodies, which are rich in RyR-containing endoplasmic reticulum but not in NMDA receptors, the fluorescence plate reader measures the mean intracellular Ca^2+^ changes in a population of CGC (Berman and Murray 2000), including dendrite-rich regions with a high density of NMDARs.

Our results confirm the recently published data of Meijer et al. (2014), who showed quantitative and qualitative differences between the results obtained using a fluorescence microscope and a fluorescence plate reader. Meijer et al. (2014) claimed that due to its higher sensitivity and reliability, single-cell fluorescence microscopy is superior to plate reader methods as a tool to assess in vitro neurotoxicity basing on intracellular Ca^2+^ recording. In our study, despite certain obvious limitations, the plate reader method, provided valid data. Of particular importance is that the results of our plate reader-based measurement of 7.5 µM TBBPA-evoked increases in intracellular Ca^2+^ levels and their sensitivity to MK-801 inhibition (Fig. 2; Table 2) were fully compatible with the results of measurements of ^45^Ca uptake in the presence of TBBPA at the same concentration (Fig. 3, see “Discussion” section below).

The measurements of ^45^Ca uptake in CGC conclusively demonstrated the participation of NMDARs in the Ca^2+^ imbalance caused by TBBPA-induced neuronal Ca^2+^ influx. TBBPA (7.5, 10, and 25 µM) concentration-dependently increased ^45^Ca uptake, which was completely abolished by MK-801. Treatment with the RyR-inhibiting mixture composed of bastadin 12 with ryanodine did not reduce this effect, but instead produced MK-801-sensitive increases in ^45^Ca uptake (Fig. 3). The effect induced by bastadin 12 plus ryanodine seems to result from short-term activation by ryanodine of the RyR channels, leading to transient increase in [Ca^2+^]_i_ (Fig. 1e). Such increase may trigger glutamate release from the neurotransmitter pool in the presynaptic terminals leading to activation of the NMDARs and MK-801-sensitive calcium entry to neurons. Stimulatory effects of TBBPA and of bastadin 12 plus ryanodine on ^45^Ca uptake in CGC were additive, suggesting that they reflect different mechanisms. The short-lived intracellular Ca^2+^ release via RyRs induced by ryanodine is unlikely to reflect neuroprotective properties attributed to upregulation of RyRs in neurons under different experimental conditions (Supnet et al. 2010).

Although the mechanism of the latter phenomenon is not well understood, it is unlikely to have anything to do with the effects of TBBPA. The main results of the ^45^Ca uptake measurements collectively indicate that in CGC, the administration of TBBPA leads to the activation of NMDARs, which is independent of TBBPA-evoked intracellular Ca^2+^ release. The results also indicate that the influx of extracellular Ca^2+^ into TBBPA-treated neurons is mediated exclusively by NMDARs. The latter finding is consistent with the data of Hendriks et al. (2012), who indicated that TBBPA inhibits voltage-gated calcium channels. Also one of the positive control PCB 95 potently increases ^45^Ca uptake in CGC (Fig. 5), which is consistent with the observation that this substance not only induces intracellular Ca^2+^ release via RyRs, but also amplifies ionotropic glutamate receptor signaling (Gafni et al. 2004).

The mechanism underlying the TBBPA-induced activation of NMDARs in CGC is unclear and may be complex. Reistad et al. (2007) attributed the TBBPA-evoked increase in [Ca^2+^]_i_ to the accumulation of glutamate and aspartate in the incubation medium, resulting from the previously described TBBPA-mediated inhibition of glutamate transporters in neuronal plasma membranes (Mariussen and Fonnum 2003). However, these authors observed such an increase in glutamate level after incubating the CGC with TBBPA for at least 90 min (Reistad et al. 2007), while TBBPA-induced increases in [Ca^2+^]_i_ and ^45^Ca uptake are immediate (Figs. 1, 2, 3). The glutamate that potentially activates NMDARs in neurons treated with TBBPA could also be released from presynaptic nerve terminals, which could be triggered by the release of intracellular Ca^2+^ via RyRs (for review see Nizami et al. 2010). However, in the present study, the TBBPA-evoked increase in ^45^Ca uptake was not inhibited by bastadin 12 with ryanodine (Fig. 3), which does not support such an explanation. Rather, this result indicates that the activation of NMDARs in CGC treated with TBBPA is independent of intracellular Ca^2+^ release.

The increase of [Ca^2+^]_i_ in cell cultures treated with TBBPA have been shown in several studies using various models (Reistad et al. 2007; Ogunbayo et al. 2008; Al-Mousa and Michelangeli 2012, 2014; Hendriks et al. 2014; Zieminska et al. 2014b). The role of RyRs in this phenomenon has been previously demonstrated (Ogunbayo et al. 2008; Zieminska et al. 2014b). Our present results add credence to the previous observations by demonstrating that bastadin 12 plus ryanodine inhibited increases in [Ca^2+^]_i_ evoked by TBBPA (Figs. 1, 2). As such, combination of the two drugs turned out to provide a pharmacological tool to determine the relative contribution of RyR-mediated intracellular Ca^2+^ release in TBBPA-evoked cytotoxicity in neurons.

We noticed that irrespective of TBBPA concentration and the platform used for Ca^2+^ measurements, simultaneous application of inhibitors of NMDAR-mediated neuronal Ca^2+^ influx and of RyR-mediated Ca^2+^ release completely abolished the increases in [Ca^2+^]_i_ evoked by TBBPA. These results indicate that NMDARs and RyRs are the only Ca^2+^ ionophores involved in TBBPA-induced Ca^2+^ transients in CGC. This finding is consistent with the data of Hendriks et al. (2012), who suggested that TBBPA strongly inhibits the activity of voltage-gated Ca^2+^ channels. Similarities of the effects of TBBPA, thapsigargin, and PCB95 on intracellular Ca^2+^ stores have been previously demonstrated (Mack et al. 1994; Gafni et al. 2004; Pessah et al. 2010; Zieminska et al. 2014b). Our present data clearly bespeak a similar potential of TBBPA and PCB 95 in inducing Ca^2+^ transients in CGC, suggesting similar underlying mechanisms, in either case comprising the transformation of RyRs into leak channels.

Collectively, our calcium studies showed that, even at low µM concentrations, TBBPA activates NMDARs and RyRs, and the activation of both of these channels participates in the increase in [Ca^2+^]_i_ in CGC. It may be tempting to speculate that the effects are positively looped: Ca^2+^ influx into neurons via the activation of NMDARs could potentiate the mobilization of Ca^2+^ from ryanodine-sensitive intracellular stores via calcium-induced calcium release, while the increase of [Ca^2+^]_i_ could trigger glutamate secretion from presynaptic nerve terminals, activating NMDARs. In so far, the present results failed to provide support to the concept, rather suggesting that the TBBPA-mediated activation of NMDARs and RyRs in CGC are two independent processes.

After identifying the mechanisms of TBBPA-induced Ca^2+^ imbalance in primary CGC cultures, we evaluated their role in the cytotoxicity of TBBPA. In the present experiments, CGC were acutely challenged with TBBPA for the measurements of both Ca^2+^ transients and cytotoxicity, the latter being assessed with a 24-h delay. Moreover, in the present experiments, the exposure of the CGC to TBBPA was performed exclusively using serum-free incubation medium because of the high hydrophobicity of TBBPA and the ease with which it binds to proteins. Our experience is that prolonged incubation or culturing of CGC under serum-free conditions is poorly tolerated by these cells. The present experimental protocol differs from that of several other studies addressing the mechanisms of TBBPA cytotoxicity, which usually examined the survival of the cells after a prolonged 18 or 24 h incubation with TBBPA, whereas TBBPA-evoked changes in intracellular Ca^2+^ levels were measured during acute exposure to this substance (Al-Mousa and Michelangeli 2012; Hendriks et al. 2012, 2014; Ogunbayo et al. 2008; Reistad et al. 2007). It is worth noting that the LC_50_ value of 12.5 µM found for TBBPA in our study (Fig. 1a) is very close to values of 7–15 µM which have been published by others (Al-Mousa and Michelangeli 2012; Reistad et al. 2007). Whether and in what degree the present protocol is congruent with the status anticipated in the in vivo setting remains to be seen.

In our experiments, staining with propidium iodide was used to evaluate the percentage of dead cells (Ankarcrona et al. 1995). Although the authors of this method originally designed it to visualize apoptosis (Ankarcrona et al. 1996), in the present study, we did not differentiate between necrotic and apoptotic neuronal death because this distinction was not the purpose of the present study. There are divergent opinions concerning TBBPA-induced apoptosis in neurons. While Reistad et al. (2007) suggested that TBBPA induces caspase-independent apoptosis in CGC, Wojtowicz et al. (2014) described TBBPA-induced caspase-3-dependent apoptosis of primary cortical cells, followed by secondary necrosis.

Our results (Fig. 4a, b) showed that an acute, 30-min exposure of CGC to 7.5 µM TBBPA was below the threshold of cytotoxicity, although it resulted in both ^45^Ca uptake and an increase in [Ca^2+^]_i_. At 10 and 25 µM concentrations, TBBPA-induced concentration-dependent cytotoxicity, which was equally and only partially reduced by the separate application of MK-801 or bastadin 12 with ryanodine. It is noteworthy that combined application of all of these inhibitors, which completely abolished TBBPA-evoked increases in [Ca^2+^]_i_, only slightly increased cytoprotection. There was also a lack of an additional increase in the rate of cytoprotection when all of these inhibitors were present during the entire 24-h CGC culturing period after the TBBPA challenge (results not shown). These data indicate that the mechanisms that trigger TBBPA cytotoxicity in CGC are complex, comprising both Ca^2+^-dependent, NMDAR- and RyR- mediated processes and Ca^2+^-independent processes.

As has been previously demonstrated by Reistad et al. (2007), MK-801 at a concentration of 3 µM provided very effective cytoprotection of the CGC incubated for 24 h in the presence of 10 µM TBBPA; incubating the cultures in Ca^2+^-free buffer provided less extensive protection from this cytotoxicity, suggesting a role of Ca^2+^ influx via NMDARs in TBBPA cytotoxicity. Our present results, which were obtained under different conditions involving the acute exposure of CGC to TBBPA and using 0.5 µM MK-801, together with new data on Ca^2+^ transients, directly confirmed the role of NMDARs in TBBPA cytotoxicity. Moreover, we confirmed the cytoprotective potential of the well-tolerated RyR-inhibiting mixture composed of bastadin 12 with ryanodine. Although the efficacy of these substances in the inhibition of intracellular Ca^2+^ release in CGC challenged with thapsigargin or TBBPA was demonstrated previously (Zieminska et al. 2006–2007, 2014b), to our knowledge, this is the first use of bastadin 12 and ryanodine in the study of the cytotoxicity of Ca^2+^ releasers in neurons. New results include the observation that the inhibition of TBBPA-induced intracellular Ca^2+^ release was as effective as the NMDAR antagonist MK-801 in providing neuroprotection and that, in addition to the Ca^2+^ -dependent mechanisms of TBBPA cytotoxicity, there are mechanisms not directly related to increases in intracellular Ca^2+^.

When focusing attention on Ca^2+^ imbalance, one should remember that oxidative stress and enhanced ROS production reportedly contribute to the mechanisms of TBBPA cytotoxicity in different experimental models, including CGC (Szymanska et al. 2000; Shi et al. 2005; Reistad et al. 2007; Zieminska et al. 2012). We tentatively propose that the putative Ca^2+^-independent production of ROS may be involved in the toxic effects of TBBPA in CGC that are resistant to the protection extended by MK-801 and bastadin 12 with ryanodine.

TBBPA is a putative environmental pollutant that is also present in biological samples (Alaee and Wenning 2002; de Wit 2002). Different BFRs and PCBs affect Ca^2+^ homeostasis and intracellular signaling in neurons (Kodavanti et al. 2005; Pessah et al. 2010; Reistad et al. 2007; Ogunbayo et al. 2008, Hendriks et al. 2012). Our present results provide new information on the role of NMDARs and RyRs in the mechanisms of Ca^2+^ imbalance and cytotoxicity in neurons that have been acutely exposed to TBBPA at micromolar concentrations. Hendriks et al. (2012) demonstrated that nicotinic acetylcholine and GABA receptors and voltage-gated calcium channels are also targets of TBBPA interactions. These data are worrying. However, caution is needed when extrapolating these in vitro results to an in vivo situation. TBBPA concentrations in human plasma do not exceed low nanomolar levels (Reistad et al. 2007), and acute poisoning with higher TBBPA doses in industry seems highly unlikely (Szymanska et al. 2000). Moreover, based on the short half-life and lack of biomagnification in the food web, TBBPA can be considered a weak environmental toxin (Darnerud 2003; Sjödin et al. 2003). However, TBBPA is an intriguing pleiotropic toxin, and studies of the mechanisms of its interference may disclose new relations between signaling pathways in neurons.

In conclusion, the results of this in vitro study directly demonstrated that in CGC, TBBPA-induced increases in [Ca^2+^]_i_ are due to the influx of Ca^2+^ ions into neurons via NMDARs and their release from intracellular stores through leaky RyRs; both of these processes, as well as other Ca^2+^-independent mechanism(s), are involved in the acute cytotoxicity of TBBPA.
